# Importance of the 5’ untranslated region for recombinant enzyme production in isolated *Bacillus subtilis* 007

**DOI:** 10.1186/s13568-025-01832-6

**Published:** 2025-02-07

**Authors:** Jana Senger, Adriana Schulz, Ines Seitl, Martin Heider, Lutz Fischer

**Affiliations:** Institute of Food Science and Biotechnology, Department of Biotechnology and Enzyme Science, University of Hohenheim, Garbenstr. 25, 70599 Stuttgart, Germany

**Keywords:** *Bacillus subtilis*, Expression, Enzymes, 5’untranslated region

## Abstract

The production of industrial enzymes requires an efficient expression system with a suitable host. This study investigated the isolated *Bacillus subtilis* 007 as a host for expressing three enzymes with potential application in the food industry. Firstly, testing the P_aprE_ and P_43_ promoters and the corresponding 5’ untranslated regions revealed great differences in the production of the recently discovered β-galactosidase from *Paenibacillus wnnyii*. Expression controlled by the P_aprE_ promoter yielded a significantly higher activity of 2515 µkat/L, compared to 56 µkat/L with the P_43_ promoter. Modifications on the P_aprE_ core promoter region or the spacer, the sequence between the Shine-Dalgarno sequence and the start codon, did not improve β-galactosidase production. Since the *aprE* 5’ untranslated region contributes to a high mRNA stability, it was incorporated into the P_43_ construct to determine whether mRNA stability is responsible for the differences observed in β-galactosidase production. Interestingly, mRNA stability was significantly improved and led to a nearly 50-fold higher β-galactosidase production of 2756 µkat/L. This strategy was successfully validated by the expression of two other enzymes: the cellobiose-2-epimerase from *Caldicellulosiruptor saccharolyticus* and the β-glucosidase from *Pyrococcus furiosus*. These findings underscored the crucial role of post-transcriptional regulation and emphasized mRNA stability as a key role in recombinant enzyme production in *B. subtilis* 007.

**Supplementary Information:**

The online version contains supplementary material available at 10.1186/s13568-025-01832-6.

## Introduction

A cost-efficient and high-yield enzyme production is a prerequisite for the extensive use of enzymes in the food industry. Microbial hosts are used in preference to animals and plants for enzyme production because of their technical and economic benefits, such as shorter production time, higher product yields and the ease of genetic modification (Robinson 2015; Guerrand 2018; Deckers et al. 2020). Food enzymes and the production host undergo an intense risk assessment by the European Food Safety Authority (Deckers et al. 2020). Host organisms with the qualified presumption of safety status are pre-assessed as safe and commonly used for production. The list of qualified presumption of safety organisms comprises filamentous fungi, such as *Aspergillus* species, yeasts, like *Komagataella phaffii*, or bacteria, such as *Bacillus subtilis* (EFSA et al. 2023).

The Gram-positive bacterium *B. subtilis* is an attractive host for recombinant protein production due to the organism’s genetic accessibility and short cultivation times on inexpensive substrates (Su et al. 2020; Stülke et al. 2023). The success of a *B. subtilis* expression system depends on the strain selected and the target protein. Thus, several studies focused on optimizing the expression conditions in *B. subtilis* to achieve efficient and high-yield production of a specific target protein (Nijland and Kuipers 2008; Cai et al. 2019).

Bacterial gene expression is primarily regulated at the transcriptional level (Deal et al. 2024), therefore, promoters have been screened or modified to enhance recombinant enzyme production in *B. subtilis* (e.g. Song et al. 2016; Han et al. 2019). However, post-transcriptional regulations, such as translation initiation, immediately impact the proteome and are crucial for protein production (Laursen et al. 2005; Duval et al. 2015). Several studies focused on improving translation initiation by modifying elements in the 5’ untranslated region (5’UTR), such as changing the ribosomal binding site (Jan et al. 2001; Pang et al. 2020). Others varied the spacer length, the distance between the Shine-Dalgarno (SD) sequence and the start codon, to optimize recombinant protein production (Vellanoweth and Rabinowitz 1992; Sauer et al. 2018; Volkenborn et al. 2020).

Moreover, the 5’UTR can influence translation efficiency by affecting the mRNA stability. Several 5’UTR in *Bacillus* have been identified possessing RNA stabilizing elements (RSE), which contribute to a long mRNA half-life within the cell (Agaisse and Lereclus 1996; Jürgen et al. 1998; Hambraeus et al. 2000; Condon 2003). These RSE prevent mRNA degradation by the formation of mRNA secondary structures, causing ribosome stalling and/or providing strong ribosome and protein binding sites (Condon 2003). Hue et al. (1995) used the RSE of the SP82 bacteriophage and improved production of the model protein LacZ in *B. subtilis* 7-fold. Similar results were reported by Phan et al. (2013), who introduced the *gsiB* 5’UTR into their constructs, which is known as a determinant for high mRNA stability (Jürgen et al. 1998; Phan et al. 2013). As a result, the recombinant production of the model protein BgaB in *B. subtilis* was enhanced 200-fold (Phan et al. 2013). Synthetic stabilizing 5’UTR can also improve recombinant enzyme production, as shown for nattokinase (EC 3.4.21.62) production in *Bacillus licheniformis* (Xiao et al. 2020).

This study investigated the isolated *B. subtilis* 007 as a host for recombinant enzyme production and determined the impact of selected expression elements on production. Three enzymes with potential applications in the food industry were chosen as target proteins. The recently discovered β-galactosidase from *Paenibacillus wynnii* (β-gal-Pw; EC 3.2.1.23) possesses remarkable properties to produce lactose-free and galacto-oligosaccharide-enriched dairy products. This enzyme, for instance, is not inhibited by the D-galactose formed and has a low K_M, Lactose_ = 0.63 mM for the substrate lactose (Fischer and Lutz-Wahl 2020; Lutz-Wahl et al. 2024). The thermostable cellobiose-2-epimerase from *Caldicellulosiruptor saccharolyticus* (CsCE; EC 5.1.3.11) catalyzes the conversion of lactose in milk to epilactose and lactulose, which act as prebiotics (Park et al. 2011; Kim and Oh 2012; Rentschler et al. 2015). Moreover, the production of the β-glucosidase from *Pyrococcus furiosus* (CelB; EC: 3.2.1.21) was investigated. This thermostable enzyme can be potentially applied for lactose hydrolysis, galacto-oligosaccharide formation and lactulose production (Petzelbauer et al. 2002; Splechta et al. 2002; Mayer et al. 2004).

## Material & methods

### Chemicals and enzymes

All chemicals were purchased from Biosolve (Valkenswaard, Netherlands), Sigma Aldrich (St. Louis, USA), Carl Roth GmbH (Karlsruhe, Germany) and Fisher Scientific (Hampton, USA). Oligonucleotides for polymerase chain reaction (PCR) and QuikChange mutagenesis were ordered from Biomers (Ulm, Germany). Restriction enzymes for cloning, Q5^®^ High-Fidelity DNA Polymerase for PCR and NEBuilder^®^ HiFi DNA Assembly for Gibson Assembly were purchased from New England Biolabs GmbH (NEB; Frankfurt am Main, Germany). The T4 DNA ligase was obtained from Thermo Fisher Scientific (Hampton, USA).

### Strains and media

*Escherichia coli* XL1 and NEB 5-alpha cells were grown in LB medium (Lennox) at 37 °C containing 80 µg mL^− 1^ 5-bromo-4-chloro-3-indolyl-beta-D-galactopyranoside (X-Gal) and 100 µg mL^− 1^ ampicillin. *B. subtilis* 007 (DSM 118688) was grown on LB-agar plates with 7.5 µg/mL neomycin and 80 µg/mL X-Gal. Shake flask cultivations were done using the media described by Zhang et al. (2020). Pre-cultures were grown in seed medium consisting of 40 g L^− 1^ sucrose, 30 g L^− 1^ soy peptone, 6 g L^− 1^ KH_2_PO_4_ and 2.04 g L^− 1^ MgCl_2_ × 6 H_2_O. Main cultivations were done using fermentation medium, which was composed of 70 g L^− 1^ sucrose, 50 g L^− 1^ soy peptone, 5 g L^− 1^ KH_2_PO_4_ and 3.06 g L^− 1^ MgCl_2_ × 6 H_2_O. Regarding the preparation of seed and fermentation medium, soy peptone was autoclaved separately, and sucrose and salts were sterilized together by filtration (Ø 0.2 μm).

### Construction of expression plasmids

Expression plasmids were constructed based on the pLF-plasmid as a vector (Senger et al. 2024; Supplemental Fig. 1A). Plasmids and primers used for cloning are listed in Supplemental Tables 1 and 2. The *β*-gal-Pw gene (Accession No: JQCR01000002.1:1461278–1464406) was used as codon-optimized sequence (Senger et al. 2024; Sequence in Supplemental Fig. 1B). The codon-optimized *β*-gal-Pw gene, the native CelB (Accession No: KF420204.1) and CsCE (Accession No: ABP65941.1) genes were integrated into the vectors possessing either P_43_ (pLF43) or P_aprE_ (pLFA) by conventional cloning. The gene sequences were amplified by PCR using the Q5^®^ High-Fidelity DNA Polymerase and primer pairs P1/P2, P3/P4 or P5/P6, respectively. The amplified inserts were purified using the DNA Clean & Concentrator Kit (Zymo Research, Orange, USA) and cloned into the vectors by *Spe*I/*Xho*I or *Spe*I/*Pac*I digestion and ligation using the T4 DNA ligase generating pLF1– pLF6 (Supplemental Table 1).

Modification of the P_aprE_ core promoter region generating pLF7 was done using the Q5 Site-directed Mutagenesis Kit (NEB; Frankfurt am Main, Germany), pLF1 as a template and primers P7 and P8. After PCR, the sample was treated with a kinase/ligase/*Dpn*I mix and 2 µL were used for the transformation of NEB 5-alpha *E. coli* cells.

The removal of nucleotides (nt) in the spacer sequence generating pLF8 was done by QuikChange mutagenesis using the Q5^®^ High-Fidelity DNA Polymerase, pLF1 as a template and the primer pair P9/P10. Two 25 µL reaction samples were prepared, which possess either P9 or P10 to prevent self-annealing. The following thermocycling conditions were used: (i) 98 °C for 30 s; (ii) 98 °C for 10 s; (iii) 62 °C for 30 s; and (iv) 72 °C for 180 s. Step (ii) to (iv) were repeated for 15 cycles. Afterwards, the program was paused for 10 min at 30 °C in step (v). Both samples were combined and 2 µL 10 mM dNTPs and 0.6 µL of Q5 polymerase were added until the program proceeded by repeating step (ii) to (iv) for another 15 cycles followed by the final elongation (vi) for 10 min at 72 °C. After PCR, the sample was treated with *Dpn*I overnight at 37 °C to remove the methylated template DNA.

Changing the *cdd* 5’UTR against the *aprE* 5’UTR sequence in pLF2, pLF4 and pLF6 was done by Gibson Assembly, generating pLF9, pLF10 and pLF11. Therefore, the P_43_ vector sequence from pLF2 was amplified with 5’ overhangs with primer pairs P11/P12 and P15/P12. The primer pairs P13/P14 or P13/P16 were used for amplification of the 5’UTR_gene sequence from pLF1, pLF3 and pLF5, respectively. The amplified fragments were purified using the GeneJet Gel extraction kit (Thermo Fisher Scientific, Hampton, USA) and assembled with the NEBuilder^®^ HiFi DNA Assembly mix. An amount of 10 µL of assembly, ligation or QuikChange sample were used for the transformation of chemically competent *E. coli* XL1 cells.

After the transformation of either *E. coli* XL1 or *E. coli* 5-alpha, the plasmid was isolated from single colonies of *E. coli* using the GeneJET Plasmid Miniprep Kit (Thermo Fisher Scientific, Hampton, USA). Correct cloning was verified by digestion and sequencing (Eurofins Genomics, Ebersberg, Germany).

### Transformation of * B. subtilis* 007

The transformation of *B. subtilis* 007 was done following a modified protocol of Vojcic et al. (2012). The SM1 medium comprised 0.2% (NH_4_)_2_SO_4_, 1.4% _2_HPO_4_, 0.6% KH_2_O_4_, 0.07% NaC_6_H_5_O_7_, 0.5% lucose, 0.02% MSO_4_ × 7 H_2_O, 0. % yeast extract and0.025% peptone. The SM2 edium was composed of 0.2% (NH_4_)_2_SO_4_, 1.4% _2_HPO_4_, 0.6% KH_2_O_4_, 0.07% NaC_6_H_5_O_7_, 0.5% lucose, 0.08% MSO_4_ × 7 H_2_O, 0. % yeast extract, 0.1% peptone and 0.05 CaCl_2_. Yeast extrct and peptone were autoclaved separately. All other components were sterile-filtered (Ø 0.2 μm), thereby removing precipitants.

*B. subtilis* 007 was streaked on LB agar plates, and incubated for 9–14 h at 37 °C. Single colonies were used for inoculation of 5 mL SM1 medium. After overnight incubation at 37 °C and 180 rpm, the pre-culture was diluted to an OD_600_ of 0.5 with fresh SM1 medium, and 5–10 mL were cultivated at 37 °C and 180 rpm. After 3 h, the volume was doubled using SM2 medium, and incubation continued for an additional 2 h at 37 °C and 180 rpm. The cells were competent for 1 h (Vojcic et al. 2012). Plasmid DNA was amplified using the Cytiva IllustraTM Templiphi Kit (Fisher Scientific, Hampton, USA) and 1 µg of DNA was added to 500 µL of competent *B. subtilis* 007 cells. After incubation for 30 min at 37 °C, 300 µL of LB medium was added, and incubation proceeded for another 30 min. Cells were plated on LB agar plates with 7.5 µg/mL neomycin and 80 µg/mL X-Gal and incubated overnight at 37 °C or at room temperature for three days. The recombinant *B. subtilis* strains are listed in Supplemental Table 3.

### Cultivation of recombinant* B. subtilis* strains

Freshly transformed *B. subtilis* cells were used for the inoculation of 10–15 mL of seed medium with 7.5 µg mL^− 1^ neomycin and incubated overnight at 37 °C and 180 rpm. The pre-culture was used for the inoculation of the main culture to an OD_600_ of 0.05. Cultivation for recombinant enzyme production was done using 150 mL fermentation medium with 7.5 µg mL^− 1^ neomycin in 1 L shake flasks at 30 °C and 110 rpm for 56 h. Sampling was done periodically to determine the OD_600_, pH, protein content and intracellular enzyme activity.

Cultivations for mRNA stability determinations were done in 40 mL fermentation medium with 7.5 µg mL^− 1^ neomycin in 300 mL shake flasks at 30 °C and 100 rpm for 48 h. After 48 h, rifampicin with a final concentration of 100 µg mL^− 1^ was added to block the transcription. Sampling was done after 0, 7.5, 15 and 30 min of rifampicin addition by centrifugation of a 10 mL culture at 13,000 × g for 10 min at 4 °C. The pellets were immediately frozen in liquid nitrogen and stored at -80 °C until used for RNA isolation and qRT-PCR analysis.

### Sample preparation and cell disruption

For sampling to determine enzyme activity and protein content, an amount of 5–10 mL cultivation sample was centrifuged at 8000 × g for 10 min at 4 °C. Regarding cell disruption, the pellet was solubilized in the respective activity buffer with 2 mg/mL lysozyme and 1 mM phenylmethylsulphonyl fluoride, preparing a 30% cell suspension. The latter was incubated for 1 h at room temperature. Cell suspensions of the *B. subtilis* strains for β-gal-Pw and CelB production were additionally mixed with 0.1–0.2 mm glass beads (Willy A. Bachofen GmbH, Switzerland) disrupted in the TissueLyser II (Qiagen, Hilden, Germany) at 30 Hz for 30 or 15 min, respectively. The cell debris was removed by centrifugation at 13,000 rpm for 10–20 min at 4 °C. The CsCE and CelB samples were additionally heated at 70 °C for 20 min and centrifuged again in the same conditions. The cell-free extract was used for activity measurements and the determination of the protein concentration according to Bradford (1976) using bovine serum albumin as a standard.

### Determination of enzyme activities

The β-galactosidase activity after cell disruption was determined as described (Mayer et al. 2004; Erich et al. 2015) using *o*NPG (*o*-nitrophenyl-β-D-galactopyranoside) as a substrate. An amount of 20 µL sample was added to a mixture of 80 µL β-gal-Pw activity buffer (100 mM potassium phosphate buffer, pH 6.75 with 5 mM MgCl_2_) and 100 µL 50 mM *o*NPG dissolved in the buffer for the determination of β-gal-Pw activity. The mixture and enzyme solution were pre-incubated separately for 5 min at 37 °C and 900 rpm and the reaction was started by adding the enzyme solution. When the sample turned yellow, the reaction was stopped by the addition of 1 M Na_2_CO_3,_ and the absorption was determined at 405 nm. The kinetic *o*NPG assay was used for the determination of CelB activity. An amount of 500 µL of CelB activity buffer (50 mM NaOAc, pH 5.0) with 600 µL *o*NPG dissolved in buffer were pre-incubated at 75 °C for 15 min. The sample was pre-incubated separately and added to the substrate-buffer mix for the start of the reaction. The absorption change was measured over a time course of 2 min. One katal was defined as the amount of enzyme that catalyzes the release of 1 mol *o*-nitrophenol from *o*NPG per second. The enzyme activity of each sample was determined in triplicate.

The isomerization activity in the CsCE samples was determined using lactose as a substrate and 50 mM PIPES at pH 7.5 with 50 mM NaCl as CsCE activity buffer. An amount of 50 µL 150 mM lactose in buffer and sample were pre-incubated separately at 80 °C and combined for the start of the reaction. After incubation at 80 °C for 10 min, the reaction was stopped by the addition of 250 mM HCl. An amount of 75 µL 220 mM NaOH and 1350 µL 50 mM PIPES pH 7.5 was added and the lactulose concentration was determined as described previously (Zhang et al. 2010). Therefore, 250 µL of sample were added to 700 µL of 75% H_2_SO_4_ and incubated for 5 min at 46 °C. Afterwards, 50 µL color-forming reagent, comprising 25 g L^− 1^ cysteine hydrochloride and 0.8 g L^− 1^ L-tryptophan in 0.01 M HCl, was added and incubated at 46 °C for 2 h. The absorption was measured at 518 nm and one katal of isomerization activity was defined as the amount of enzyme required to form 1 mol of lactulose per second.

### SDS PAGE

The cytoplasmic proteome of recombinant *B. subtilis* was visualized by sodium dodecyl sulfate polyacrylamide gel electrophoresis (SDS PAGE) using an 8 or 12.5% separating gel (Laemmli 1970). An amount of 5 µg of protein was mixed with 5 × loading buffer (0.02% (w/v) Tris-Cl, 6% (w/v) glyceol, 0.1% (w/v) bromohenol blue, 4% (w/v) SDS ad 2% (w/v) β-meraptoethanol) and loaded onto the gel. As a reference, an amount of 5 µL of protein molecular weight marker Color Precision Plus Protein™ Unstained protein standard (1610363, Bio-Rad, CA, USA) was used. Protein visualization was done by utilizing Coomassie Brilliant Blue G-250 staining (Fairbanks et al. 1971).

### qRT-PCR analysis for determination of mRNA stability

RNA isolation was done using the innuSPEED Bacteria/Fungi RNA Kit (Analytik Jena; Germany) and a modified cell disruption protocol: The pellets were resuspended in 1 mL of RL solution. A 2 mL sample was mixed with 1.5 g 0.1–0.2 mm glass beads and disrupted in the TissueLyser II at 30 Hz for 15 min. The RNA was isolated and an amount of 10 µL (< 10 ng) was treated with 1 µL DNaseI (NEB; Germany) for 15 min at 37 °C. The samples were purified using the RNA Clean & Concentrator kit (Zymo Research, Orange, CA). A two-step method was used for qRT-PCR. Firstly, 10 ng of RNA was used for cDNA synthesis using the LunaScript^®^ RT SuperMix Kit (NEB; Germany). Secondly, an amount of 2 µL was then used for qPCR in the pTOWER2.2 (Analytik; Jena, Germany). The Luna^®^ Universal qPCR Master Mix (NEB; Germany) and primer pair P17/P18 (Supplemental Table 2) were used. The mRNA stability was determined and calculated as described (Ratnadiwakara and Änkö 2018).

### In silico and statistical analyses

The translation initiation rate (TIR) was calculated using the RBS Calculator v2.1 (Salis et al. 2009). Excel was used for calculations and standard deviation was used for data evaluation. Statistical analyses were performed using the ANOVA and the t-test in SPSS (SPSS Inc., Chicago, USA). All experiments were done at least in triplicate with three independent measurements.

## Results

### Altering expression for β-galactosidase production in * B. subtilis* 007

**Fig. 1:**
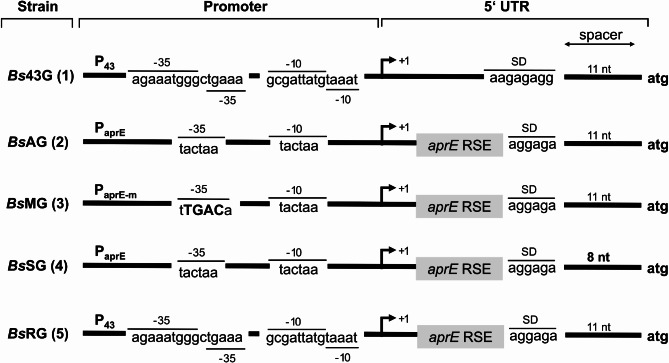
Regulatory regions and corresponding strain designation of recombinant * B. subtilis* used for β-gal-Pw production. The *B. subtilis* (*Bs*) strains were named after the element changed in the promoter region or the 5’UTR (43 = P_43_, A = P_aprE_, M = Modified P_aprE_, S = spacer and R = included RSE). The start codon of β-gal-Pw gene (G) is represented by atg. RSE = RNA stabilizing element; SD = Shine-Dalgarno sequence; 5’UTR = 5’ untranslated region

The isolated strain *B. subtilis* 007 was investigated to produce the β-galactosidase of *P. wynnii* (β-gal-Pw). Five recombinant strains were constructed differing in the promoter and 5’UTR to find the best expression conditions in *B. subtilis* 007 (Fig. 1).

Firstly, the P_aprE_ and P_43_ promoters, including the respective native 5’UTR, were tested. Both promoters are well-described and exhibit the highest activity at the transition and early stationary phase (Wang and Doi 1984; Jan et al. 2000). The β-gal-Pw gene was integrated into the vectors possessing P_43_ or P_aprE_ and the transformation of *B. subtilis* 007 resulted in the recombinant strains (1) *Bs*43G and (2) *Bs*AG (Fig. 1; Supplemental Table 3). In addition, an empty plasmid was used as a negative control.

**Fig. 2:**
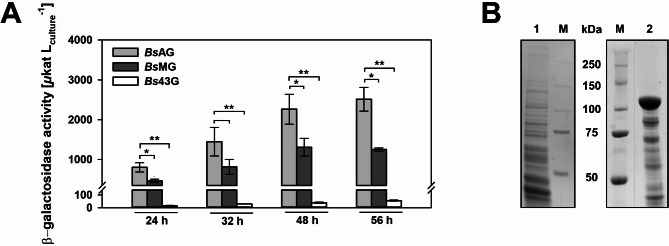
Impact of promoters on β-gal-Pw production analyzed by activity measurements (**A**) and SDS PAGE (**B**). β-galactosidase activities were determined after 24, 32, 48 and 56 h of cultivation of the recombinant *B. subtilis* strains. Stars indicate significance in the activities compared with * = *p* < 0.05, ** = *p* < 0.01. (B) SDS PAGE of the cell-free extract after 56 h of cultivation of 1 = *Bs*43G and 2 = *Bs*AG. An amount of 5 µg protein was applied. M = Marker

*Bs*43G and *Bs*AG were cultivated in shake flask cultivations for 56 h. Regarding *Bs*AG, the β-gal-Pw activity increased continuously to 2,515 ± 297 µkat/L after 56 h (Fig. 2A). Contrarily, *Bs*43G reached a significantly lower β-gal-Pw production of 56 ± 5 µkat/L indicating difficulties in expression, which was supported by the lower growth of this strain (Supplemental Fig. 2). β-galactosidase activities of only < 2 µkat/L were detected for the negative control. Thus, a strong impact of the native β-galactosidases of *B. subtilis* 007 was excluded.

The contrasting results in β-gal-Pw production using the different promoters were supported by SDS PAGE of the proteome (Fig. 2B). A strong 120 kDa band corresponding to the β-gal-Pw molecular weight demonstrated the efficient expression in *Bs*AG (2). Only a slight band was present for *Bs*43G (1).

Further improvement of transcription was targeted through modifications in the P_aprE_ core promoter region. Jan et al. (2001) showed a 100-fold increase in *lacZ* expression when the − 35 region of the P_aprE_ promoter was changed to the consensus sequence of the σ-factor A. Thus, the − 35 region was modified similarly in this study to investigate the impact on β-gal-Pw production, generating (3) *Bs*MG (Fig. 1). *Bs*MG was cultivated in shake flask cultivations and the β-gal-Pw activity was analyzed. The β-gal-Pw production was lower compared to *Bs*AG and the highest activity of 1,305 ± 223 µkat/L was reached after 48 h (Fig. 2A). In contrast to the literature, P_aprE_ modification did not improve the recombinant enzyme production in this study.

### Modifications of the 5’UTR for improved β-gal-Pw production

**Fig. 3:**
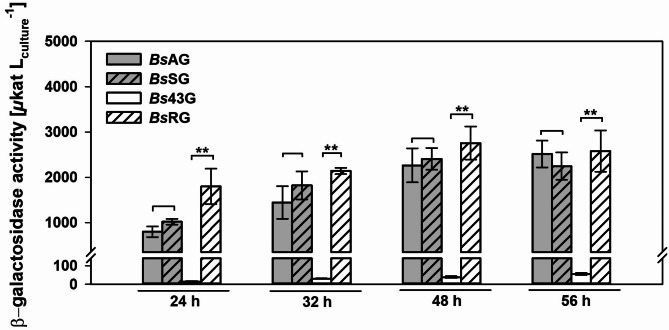
Impact of 5’UTR modifications on β-gal-Pw production. The β-galactosidase activity was determined after 24, 32, 48 and 56 h of cultivation of the recombinant *B. subtilis* strains. Stars indicate significance in the activities compared with ** = *p* < 0.01

Several elements in the 5’UTR, such as the SD sequence or the spacer length, can influence translation initiation and thus, protein production (Duval et al. 2015). The spacer is the sequence between the SD sequence and the start codon. In *Bs*AG, the spacer has a length of 11 nt. Since an optimal spacer of 7–9 nt is recommended for protein production in *B. subtilis* (Vellanoweth and Rabinowitz 1992; Volkenborn et al. 2020), three nucleotides of the spacer were removed by QuikChange mutagenesis. The generated strain (4) *Bs*SG (Fig. 1) showed a similar β-gal-Pw expression and growth behavior to *Bs*AG (Fig. 3; Supplemental Fig. 2). The maximum activity of 2,247 ± 303 µkat/L was detected after 56 h, which was not significantly lower than in *Bs*AG. In contrast to the literature, the spacer length did not affect β-gal-Pw production in this study.

In addition, RSE in the 5’UTR were shown to improve the recombinant enzyme production in *B. subtilis* by enhancing the mRNA stability (Condon 2003; Hohmann et al. 2017). The 5’UTR of the extracellular alkaline protease AprE comprises such RSE (Hambraeus et al. 2000, 2002), which are also contained in the 5’UTR of the P_aprE_ constructs used in this study. By contrast, the construct comprising the P_43_ promoter lacks RSE in the 5’UTR (Fig. 1). It was hypothesized that the low β-gal-Pw production in *Bs*43G is due to less stable mRNA, resulting in lower translation efficiency. The 5’UTR of *Bs*43G was replaced by the 5’UTR of *Bs*AG generating strain (5) *Bs*RG (Fig. 1). Cultivation of *Bs*RG showed a significant increase in β-gal-Pw production compared to *Bs*43G (Fig. 3; Supplemental Fig. 3). A maximum activity of 2,756 ± 364 µkat/L was detected after 48 h, which was almost 50-fold higher than the β-gal-Pw production using *Bs*43G. A similar β-gal-Pw expression of *Bs*RG and *Bs*AG indicated that the mRNA stability, and not promoter strength, determines the β-gal-Pw production in *B. subtilis* 007.

### Enhanced translation efficiency by improved mRNA stability

**Fig. 4:**
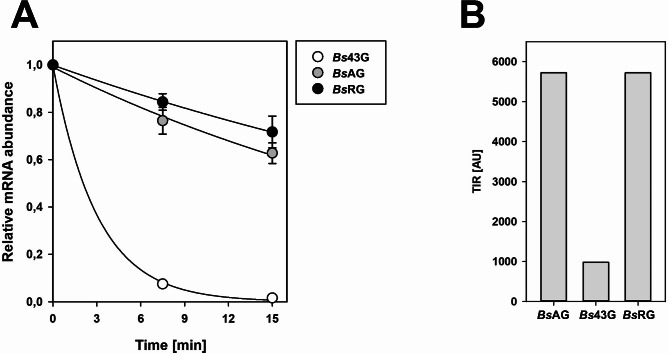
The half-life of β-gal-Pw mRNA (**A**) and TIR (**B**) in the recombinant *B. subtilis* strains. (**A**) The quantity of β-gal-Pw mRNA was determined after 0, 7.5 and 15 min of rifampicin addition by qRT-PCR. (**B**) The translation initiation rate (TIR) was calculated *in silico* using the RBS Calculator v2.1 (Salis et al. 2009). AU = Arbitrary Units

The higher β-gal-Pw production in *Bs*RG compared to *Bs*43G indicated an enhanced translation efficiency through the improved stability of the β-gal-Pw mRNA. Therefore, the β-gal-Pw mRNA stability in the different strains was determined. Transcription in the cells was blocked by rifampicin treatment followed by the quantification of the mRNA *via* qRT-PCR. After 7.5 min of rifampicin addition, the amount of β-gal-Pw mRNA in *Bs*43G was strongly reduced to less than 10% of the starting point (Fig. 4A). The half-life was determined at t_1/2_ = 2 min. By contrast, the β-gal-Pw mRNA in *Bs*RG was more stable and showed a half-life of more than 15 min, similar to the transcript stability determined in *Bs*AG (Fig. 4A).

In addition, the mRNA stability and β-gal-Pw activities measured corresponded to the *in silico* calculated TIRs (Fig. 4B). When comparing the different *B. subtilis* strains, the highest TIR was determined for *Bs*AG and *Bs*RG, both possessing the *aprE* 5’UTR. Contrarily, the TIR in *Bs*43G was remarkably lower compared to all other constructs, indicating a less efficient translation initiation. In conclusion, the 5’UTR appears to determine the β-gal-Pw production post-transcriptionally by altering the mRNA stability and translation efficiency.

### Proof of strategy for CelB and CsCE production

**Fig. 5:**
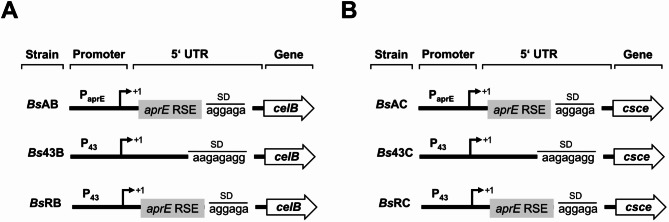
Regulatory regions and the corresponding strain designation of recombinant *B. subtilis* used for CelB (**A**) or CsCE (**B**) production. The *B. subtilis* (*Bs*) strains were named after the element changed in the promoter region or the 5’UTR (43 = P_43_, A = P_aprE_ or R = included RSE). The start codon of β-gal-Pw gene (G) is represented by atg. RSE = RNA stabilizing element; SD = Shine-Dalgarno sequence; 5’UTR = 5’ untranslated region

The β-gal-Pw production in *B. subtilis* 007 was impacted by the 5’UTR selected and corresponding mRNA stability. To determine whether this also applies to other enzymes, the production of the β-glucosidase from *P. furiosus* (CelB) and the cellobiose-2-epimerase from *C. saccharolyticus* (CsCE) was tested in *B. subtilis* 007. Therefore, recombinant *B. subtilis* 007 strains were constructed differing in promoter and 5’UTR sequence (Fig. 5).

**Fig. 6:**
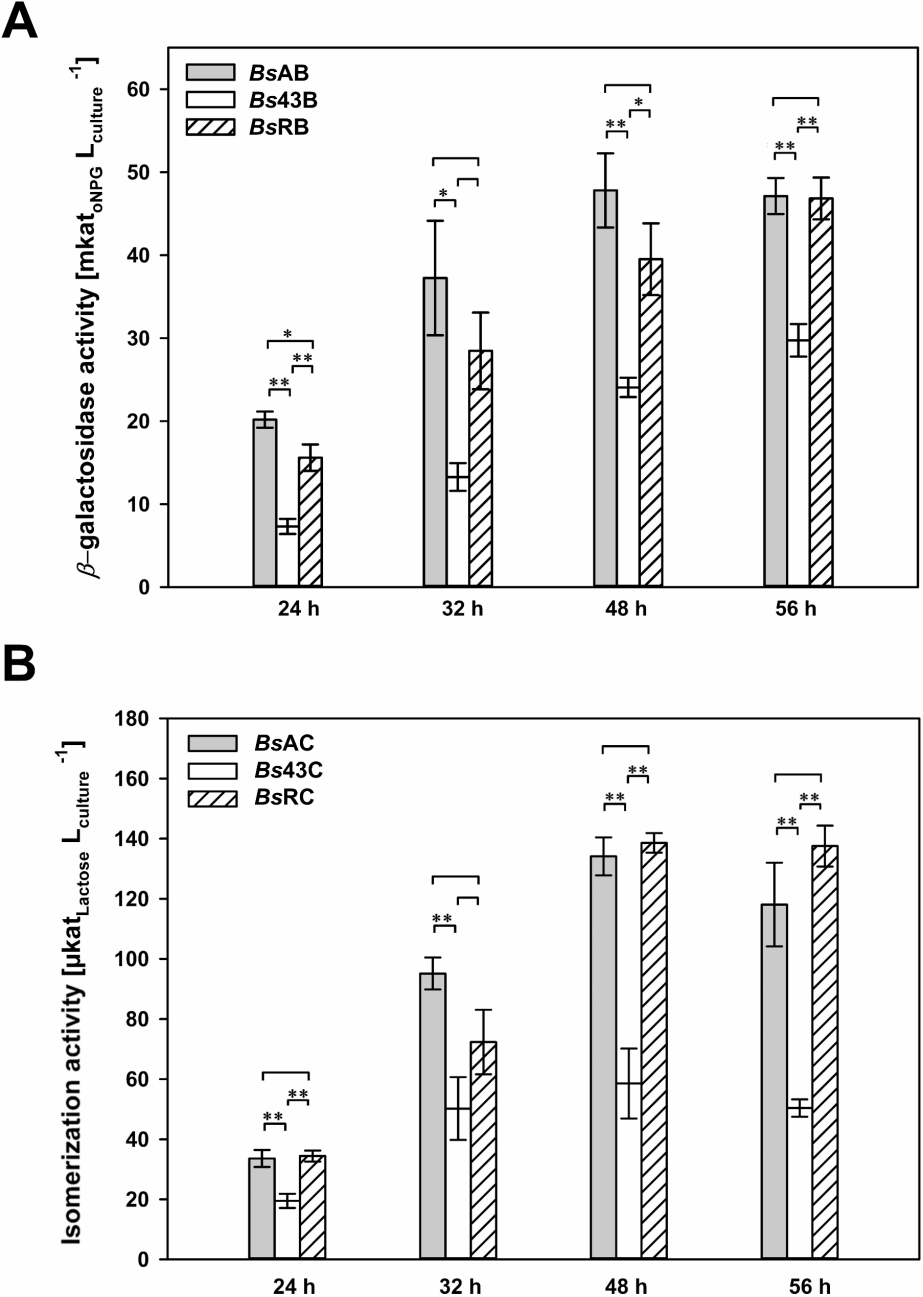
Impact of promoter and 5’UTR modification on the CelB (**A**) and CsCE (**B**) production. The intracellular activities of CelB (**A**) and CsCE (**B**) were determined after 24, 32, 48 and 56 h of cultivation of the recombinant *B. subtilis* strains. Stars indicate significance in the activities compared with * =* p* < 0.05, ** =* p* < 0.01. RSE = RNA stabilizing element; 5’UTR = 5’ untranslated region

The recombinant strains were cultivated in shake flask cultivations and the intracellular CelB or CsCE activity was determined, respectively (Fig. 6; Supplemental Fig. 4).

Firstly, the P_aprE_ and P_43_ promoters, including the corresponding 5’UTR, were compared. The P_aprE_ promoter led to a higher expression of both enzymes than the P_43_ promoter (Fig. 6). The highest CelB activity of 48 ± 5 mkat/L in *Bs*AB and CsCE activity of 134 ± 1 µkat/L in *Bs*AC were reached. By contrast, CelB production in *Bs*43B with the P_43_ promoter was consistently lower, with a maximum activity of 30 ± 2 mkat/L after 56 h. Similarly, CsCE production in *Bs*43C was more than 2-fold lower, reaching a maximum of 58.6 ± 12 µkat/L after 48 h. To evaluate the impact of the RSE on the CelB and CsCE production, the 5’UTR in the P_43_ expression plasmids was replaced by the *aprE* 5’UTR, resulting in strains *Bs*RB and *Bs*RC (Fig. 5). The *aprE* 5’UTR significantly improved the expression when controlled by the P_43_ promoter (Fig. 6). Activities of 46.8 ± 3 mkat/L in *Bs*RB and 136.6 ± 3 µkat/L in *Bs*RC were reached. Both were comparable to the activities achieved with the P_aprE_ promoter (Fig. 6). Again, the activities could also be increased for two other enzymes by introducing the *aprE* 5’UTR in the P_43_ constructs. This indicates that the 5’UTR strongly influenced the enzyme production yield in *B. subtilis* 007 at the post-transcriptional level.

## Discussion

Most of the knowledge about recombinant protein production in *B. subtilis* has been derived from the domesticated strain *B. subtilis* 168 and its derivates, which are widely used in academia and industry (Zeigler et al. 2008). These strains have adapted to laboratory life and developed increased natural competence, which simplifies genetic modifications (Zeigler et al. 2008; She et al. 2020). The adaption also resulted in the loss of ancestral traits, such as biofilm formation and lipopeptide production (McLoon et al. 2011), which is favorable for cultivation. However, undomesticated *B. subtilis* isolates are becoming increasingly attractive for recombinant protein production by often providing favorable growth properties or increased productivity (Kabisch et al. 2013; Zhang et al. 2016). The undomesticated *B. subtilis* 007 was investigated for recombinant enzyme production in this study. This strain was isolated from compost and demonstrated more stable growth compared to *B. subtilis* 168 derivates (Supplemental Fig. 5). The challenge of poor transformation ability, often seen for undomesticated *B. subtilis* strains (Nijland et al. 2010; She et al. 2020), was addressed using concatemeric plasmid DNA.

Three enzymes with a potential application in the food industry: β-gal-Pw, CelB and CsCE, were recombinantly produced in *B. subtilis* 007. Varying promoter and 5’UTR elements aimed to determine the expression conditions significantly impacting enzyme production. Notably, the use of two distinct promoters along with the corresponding 5’UTR resulted in significant differences in production. The P_aprE_ promoter led to a higher expression than the P_43_ promoter for all three enzymes. Contrarily, other studies reported a higher promoter strength of P_43_ over P_aprE_ (Kim et al. 2008; Song et al. 2016), highlighting the importance of testing different promoters to find the best transcription conditions for a specific target enzyme.

Modifying the − 35 region of the core P_aprE_ promoter to match the consensus sequence recognized by the σ-factor A (Haldenwang 1995) was intended to increase the RNA polymerase binding and transcription efficiency. However, these modifications in *Bs*MG did not improve the β-gal-Pw expression in *B. subtilis* 007, which contrasts with the results observed by Jan et al. (2001) for the *E. coli* β-galactosidase LacZ. A strong overexpression of proteins in *E. coli* can result in the breakdown of rRNAs, leading to the loss of ribosomes and the protein biosynthesis capacity (Dong et al. 1995). Similar effects might contribute to the lower β-gal-Pw production observed in this study when modifying the − 35 region of P_aprE_.

Nevertheless, the determinant for recombinant enzyme production in *B. subtilis* 007 appears to be the mRNA stability rather than transcription efficiency. When the *aprE* 5’UTR sequence was introduced into the P_43_ constructs, the mRNA stability was significantly increased, leading to enhanced production of all three enzymes tested. A nearly 50-fold increase in intracellular activity was observed for β-gal-Pw expression. Previous studies used the *gsiB* or SP28 5’UTR to improve the mRNA stability and recombinant enzyme production in *B. subtilis* (Phan et al. 2013; Hue et al. 1995). However, these studies were limited to the model proteins BgaB or LacZ and employed well-established and domesticated *B. subtilis* strains. Moreover, the crucial role of the mRNA stability in recombinant enzyme production by eliminating transcriptional differences was not demonstrated.

The *aprE* 5’UTR is known to contribute to high mRNA stability through the formation of a stem-loop structure at the 5’ end of the mRNA. Removal of the stem-loop reduced the mRNA stability from > 25 min to about 5 min (Hambraeus et al. 2000, 2002). Secondary structures in the 5’UTR can prevent 5’ ribonuclease attack, thus, affecting the mRNA abundance (Durand et al. 2015). Additionally, the SD sequence within the *aprE* 5’UTR was shown to be crucial for the mRNA stability (Hambraeus et al. 2002). Since the strength of the SD sequence itself contributes to an efficient translation initiation (Hohmann et al. 2017; Samatova et al. 2021), the changed SD in *Bs*RG might also promote the recombinant enzyme production in this study.

Another element influencing translation initiation is the spacer length between the SD sequence and the start codon. The spacer length was adjusted to the optimal length described for *B. subtilis* to improve the β-gal-Pw production in *Bs*AG (Vellanoweth and Rabinowitz 1992; Volkenborn et al. 2020). Only minor effects were observed. Volkenborn et al. (2020) showed that the differences in expression when using 8 or 11 nt were marginal in most cases, which is consistent with the results in this study. However, they also demonstrated that the optimal spacer length varies depending on the respective N-terminal coding sequence (NCS). Different NCSs resulted in alterations of the optimal spacer length for cutinase (EC 3.1.1.74) and expansin production (Volkenborn et al. 2020). The NCS probably affects the translation efficiency (Bentele et al. 2013), and engineering the β-gal-Pw NCS could further improve β-gal-Pw production in *B. subtilis*, as has been shown in several studies (Tian et al. 2019; Xu et al. 2021; Wang et al. 2022). Xu et al. (2021), for instance, rationally designed the NCS for pullulanase secretion and achieved a 515% increase in production.

This study demonstrates the key role of the 5’UTR and the associated mRNA stability for recombinant enzyme production in the undomesticated *B. subtilis* 007. It highlights the importance of modifying expression at the post-transcriptional level and provides a basis for further optimization of enzyme production in this host.

## Electronic supplementary material

Below is the link to the electronic supplementary material.

Supplementary Material 1

## Data Availability

All data generated or analyzed during this study are included in this published article [and its supplementary information files].

## Abbreviations

*B. subtilis*: *Bacillus subtilis*
β-gal-Pw: β-galactosidase from *Paenibacillus wynnii*
CelB: β-glucosidase from *Pyrococcus furiosus*
CsCE: Cellobiose-2-epimerase from *Caldicellulosiruptor saccharolyticus*
*E. coli*: *Escherichia coli*
NCS: N-terminal coding sequence
Nt: Nucleotides
*o*NPG: *o*-Nitrophenyl-β-D-galacto pyranosid
OD_600_: Optical density at 600 nm
PCR: Polymerase chain reaction
RNAP: RNA Polymerase
RSE: RNA stabilizing elements
SD: Shine-Dalgarno
SDS PAGE: Sodium dodecyl-sulfate polyacrylamide gel electrophoresis
TIR: Translation initiation rate
qRT: Quantitative reverse transcription
5’UTR: 5’ untranslated region
