# Supplementary material for: Importance of the 5’ untranslated region for recombinant enzyme production in isolated Bacillus subtilis 007
Source: AMB Express. 2025 Feb 7;15:24. doi: 10.1186/s13568-025-01832-6 (PMC11805744; doi:10.1186/s13568-025-01832-6)
Supplement: Supplementary file 1 — Supplementary Material 1 [file 13568_2025_1832_MOESM1_ESM.docx]

*Supplemental Material*

**Importance of 5’ untranslated region for recombinant enzyme production in isolated *Bacillus subtilis* 007**

Jana Senger, Adriana Schulz, Ines Seitl, Martin Heider, Lutz Fischer*

University of Hohenheim, Institute of Food Science and Biotechnology,

Department of Biotechnology and Enzyme Science, Garbenstr. 25, 70599 Stuttgart, Germany

*Corresponding author:

E-mail address: lutz.fischer@uni-hohenheim.de

Tel.: +49 711 459 22311

**Supplemental Table 1: Plasmids used in this study.**

| **Plasmid** | **Description** | **Purpose** |
| --- | --- | --- |
| pLFA | pLF-plasmid possessing *aprE* regulatory region (P_aprE_ promoter and *aprE* 5’ UTR) | Vector with P_aprE_ |
| pLF43 | pLF-plasmid possessing *cdd* regulatory region (P_43_ promoter and *cdd* 5’ UTR) | Vector with P_43_ |
| pGal_opt_ | Codon-optimized β-gal-Pw gene sequence | Template for PCR |
| pETCelB | Native CelB gene sequence | Template for PCR |
| pETCsCE | Native CsCE gene sequence | Template for PCR |
| pLF1 | β-gal-Pw gene under control of P_aprE_ with *aprE* 5’ UTR | Expression plasmid |
| pLF2 | β-gal-Pw gene under control of P_43_ with *cdd* 5’ UTR | Expression plasmid |
| pLF3 | CelB gene under control of P_aprE_ with *aprE* 5’ UTR | Expression plasmid |
| pLF4 | CelB gene under control of P_43_ with *cdd*  5’ UTR | Expression plasmid |
| pLF5 | CsCE gene under control of P_aprE_ with *aprE* 5’ UTR | Expression plasmid |
| pLF6 | CsCE gene under control of P_43_ with *cdd*  5’ UTR | Expression plasmid |
| pLF7 | β-gal-Pw gene under control of modified P_aprE_ (= P_aprE-m_) with *aprE* 5’ UTR | Expression plasmid |
| pLF8 | β-gal-Pw gene under control of P_aprE_ with *aprE* 5’ UTR and modified spacer length | Expression plasmid |
| pLF9 | β-gal-Pw gene under control of P_43_ with *aprE* 5’ UTR | Expression plasmid |
| pLF10 | CelB gene under control of P_43_ with *aprE*  5’ UTR | Expression plasmid |
| pLF11 | CsCE gene under control of P_43_ with *aprE*  5’ UTR | Expression plasmid |

**Supplemental Table 2: Primers used in this study.**

| **Primer** | **Sequence (5’🡪3’)** | **Application** |
| --- | --- | --- |
| P1 | ccactagtatgcgtaaaaaacttgtc | Forward primer for PCR of β-gal-Pw gene with *Spe*I site |
| P2 | ccctcgagttattacaccattctaatcg | Reverse primer for PCR of β-gal-Pw gene with *Xho*I site |
| P3 | gtctcgagcctactttcttgtaac | Forward primer for PCR of CelB gene with *Spe*I site |
| P4 | cgcactagtatggatatcaccc | Reverse primer for PCR of CelB gene with *Xho*I site |
| P5 | cgcactagtatggatattacaaggtttaag | Forward primer for PCR of CsCE gene with *Spe*I site |
| P6 | cttaattaacttagtcaaccctttttattatc | Reverse primer for PCR of CsCE gene with *Pac*I site |
| P7 | tcaaaaaaatgggtcttgacaaatattattccatc | Q5 Site-Directed Mutagenesis PCR of P_aprE_ -35 region in pLF1 |
| P8 | gatgattttatctctatttaggtatatcatctc | Q5 Site-Directed Mutagenesis PCR of P_aprE_ -35 region pLF1 |
| P9 | ctgaatttttttaaaaggagagggctagtatgcgtaaaaaacttg | QuikChange PCR of spacer sequence in pLF1 |
| P10 | caagttttttacgcatactagccctctccttttaaaaaaattcag | QuikChange PCR of spacer sequence in pLF1 |
| P11 | gcctcatgtttatcgttgaag | Forward primer for PCR of P_43__vector sequence for Gibson Assembly |
| P12 | cttaaaagactattctgtgaatttatattttacataatcgcgcgc | Reverse primer for PCR of P_43__vector sequence with *aprE* leader overhang for Gibson Assembly |
| P13 | gcgattatgtaaaatataaattcacagaatagtcttttaagtaag | Forward primer for PCR of β-gal-Pw sequence with P_43_ overhang for Gibson Assembly |
| P14 | cttcaacgataaacatgaggc | Reverse primer for PCR of β-gal-Pw sequence for Gibson Assembly |
| P15 | gtatccgctcatgagacaataac | Forward primer for PCR of P_43__vector sequence for Gibson Assembly |
| P16 | gttattgtctcatgagcgg | Reverse primer for PCR of gene (CelB; CsCe) sequence for Gibson Assembly |
| P17 | cagctttcaaggtgtggagtctg | Forward primer for qRT-PCR for  β-gal-Pw amplification |
| P18 | cagctttcaaggtgtggagtctg | Reverse primer for qRT-PCR for  β-gal-Pw amplification |

**Supplemental Table 3: Recombinant B. subtilis 007 strains generated in this study.**

| **Designation** | **Plasmid** | **Promoter** | **5’ UTR** | **Gene** |
| --- | --- | --- | --- | --- |
| *Bs*AG | pLF1 | P_aprE_ | *aprE* | β-gal-Pw |
| *Bs*43G | pLF2 | P_43_ | *cdd* | β-gal-Pw |
| *Bs*MG | pLF7 | P_aprE_ with modified  -35 region (= P_aprE-m_) | *aprE* | β-gal-Pw |
| *Bs*SG | pLF8 | P_aprE_ | *aprE* with modified spacer length | β-gal-Pw |
| *Bs*RG | pLF9 | P_43_ | *aprE* | β-gal-Pw |
| *Bs*C | pLFA | P_aprE_ | *aprE* | None (control) |
| *Bs*AB | pLF3 | P_aprE_ | *aprE* | CelB |
| *Bs*43B | pLF4 | P_43_ | *cdd* | CelB |
| *Bs*RB | pLF10 | P_43_ | *aprE* | CelB |
| *Bs*AC | pLF5 | P_aprE_ | *aprE* | CsCE |
| *Bs*43C | pLF6 | P_43_ | *cdd* | CsCE |
| *Bs*RC | pLF11 | P_43_ | *aprE* | CsCE |

**B**

**A**

atgcgtaaaaaacttgtctattcacctcctacaaacgggtacccggaatggaataataatcctgaatgttttcagatcaaccgaatggatgcccatgctacatggattccttttaatacaacagaggatgccttgcttggagatccacaatccagccctaactatttgtctttgaatgggatgtggaaattcgcctatgcagagactccggatcaaagaattcgtaacttttttgaaaaaaactatgattgcagttcttgggctgaactgaacgttccttctcattggcagatgcatggatatgactaccctcagtatacgaatgtaagatatccttggagtgaacgtgagccggagctcaaaccgccttttgctcctacacaatataatccggttgggtcttatgtacgaacattctctgtaccggaggactggagcgggaagcctgttttcatcagctttcaaggtgtggagtctgccttctatgtttggctcaatggagagttggtcggatacagcgaggatacattcacacctgcggaatttgacttaactccttatcttatcccgggagacaataagcttgccgttgaagtgtatcgttggtgtgacgccagttggctggaggatcaggatttctggagacttagcggcatttttagagatgtctatctttatacaacaccggaagcccatatttatgatttctttgttcgaacagaactggatgagcagtacagacatgctgaattgcagctggatgtgaagcttatggattattttgaaagaacagctgaggcagttattgtgcatgcgcagctttatgatcacgatcagaacgctatctttgatcagccgctttcacagacagtctacttcaatagcgcttctacacagacgctccaattctcttcttctattattgacccgttaaaatggagtgcggaacatccgaatctttatacgttagtgttgtctttacatcatgtggatggagagctcatggaagccgttagctgccgtgttggctttcgtacattcgagcttaaggacggtttaatgaagattaatggaaaaagaatcgtctttaagggcgtgaacagacatgaattctcatgcgatacaggcagatcaattgatgtcgatgatatggtaagagacattcttctgatgaaagcacacaacatcaatgccgttagaacttctcattatccgaatcagacaatttggtatgacctttgtgaccaatatgggctgtatgtaattgatgagacaaatctggagacacatggttcttggagctatggacagacagacttaggcggaaatacggtacctggcagtagacctgaatggagagcgaatgtactggatagatgtaattcaatgctccaaagagacaaaaaccacccttccatcgtcatctggtcactcggtaacgagtcctttggcggtgataactttgtggctatgcatgattttcttaagaaagaagacccttccagacttgtccattatgaaggcctctttcactatagagagagcgatgttgcctctgacattgagagcacaatgtacattagtccggccgatgtggaacagtatgcgcttaatgatccaaagaagccttatattctttgcgagtatagccatgcaatgggcaactcctgcgggggattgcatctttactgggaagtttttgagaaatatgatattttacagggtgcctttatctgggactggattgatcaagcgattcgacttaagcaagccgacggatccatgcatatggcctacggcggagacttcggggaatcccctcatgacggcaacttctgcggaaatggcttgattttcgctgaccgttcagtatctcctaagctgtatgaggtcaagaaatgctaccagaatgttaaatttgaggctgtcgatttagagcgaggcatttatagagtaacaaatcagaatctttttacggatttggcggagtacgcactggcttgggaagtaagctgtaacggcaatcctgtgcttaagggtactgttgatctggctgtccctgcaggagaatctgcagaaatttctgttcctgtggtcgatgaacctaacctgcaatccgagggcgagcatgtcctcacgtttagcctgcaattgaagaaatccacactttgggcagacgcaggccatgaagtggcatgggaacaattcctgctgcctacacctcagtttatggcgggtcaggatcaagactctgttttgacatcagatcgtggtgtgattgtggaggaacaagcagggcgtttaactgttcaggcggcggatgtctcccttcaattcagtacttcaagcggttatctgatctctatgcaaaataaaggcaaggaactgctgctcgaacctgttcgtccgaatttctggcgtgcagttacggataacgatcttgggaacaaacaccatgagcgttgcgcggtttggaatacagcgggagctggctgtacactagcttcctttgaatctcataagaatgtagatggcgtaactgttcgtgctaagtacacagtacctacagtacctatttcttcactgattttggaatatagaatccaagaaaatggctccattgaagtctttgaagagctttcaccgggcatgggcttgccggagattcctgaaattggcctcatgtttatcgttgaagatcgactcgacacagtttcatggtatggcagaggaccgcatgagaattactgggacagaaagacaggcgccagacttggatatttttctggaagtgtccaggatcagttcgttccgtatattagacctcaagaatgcggaaacaaaacggatgtgcgttttgccagtatcactgggggtattaacggctcaggattccgtgtggatggcgatcctgtactggaacttaatgccttgccttggacacctgctgagttggaagcgaatgatcacatttataagctgccagccagtaataaaacagttgtgcgtgtgaactacaagcaaatgggtgtaggtggagacaacagctggggcgcgacgacacatcctgaattcactttgccggcggatcaaacttatgggtttagatttacgattagaatggtgtaa

**Supplemental Figure 1: Plasmid map of pLFA (A) and β-gal-Pw sequence codon-optimized for *B. subtilis* (B).**


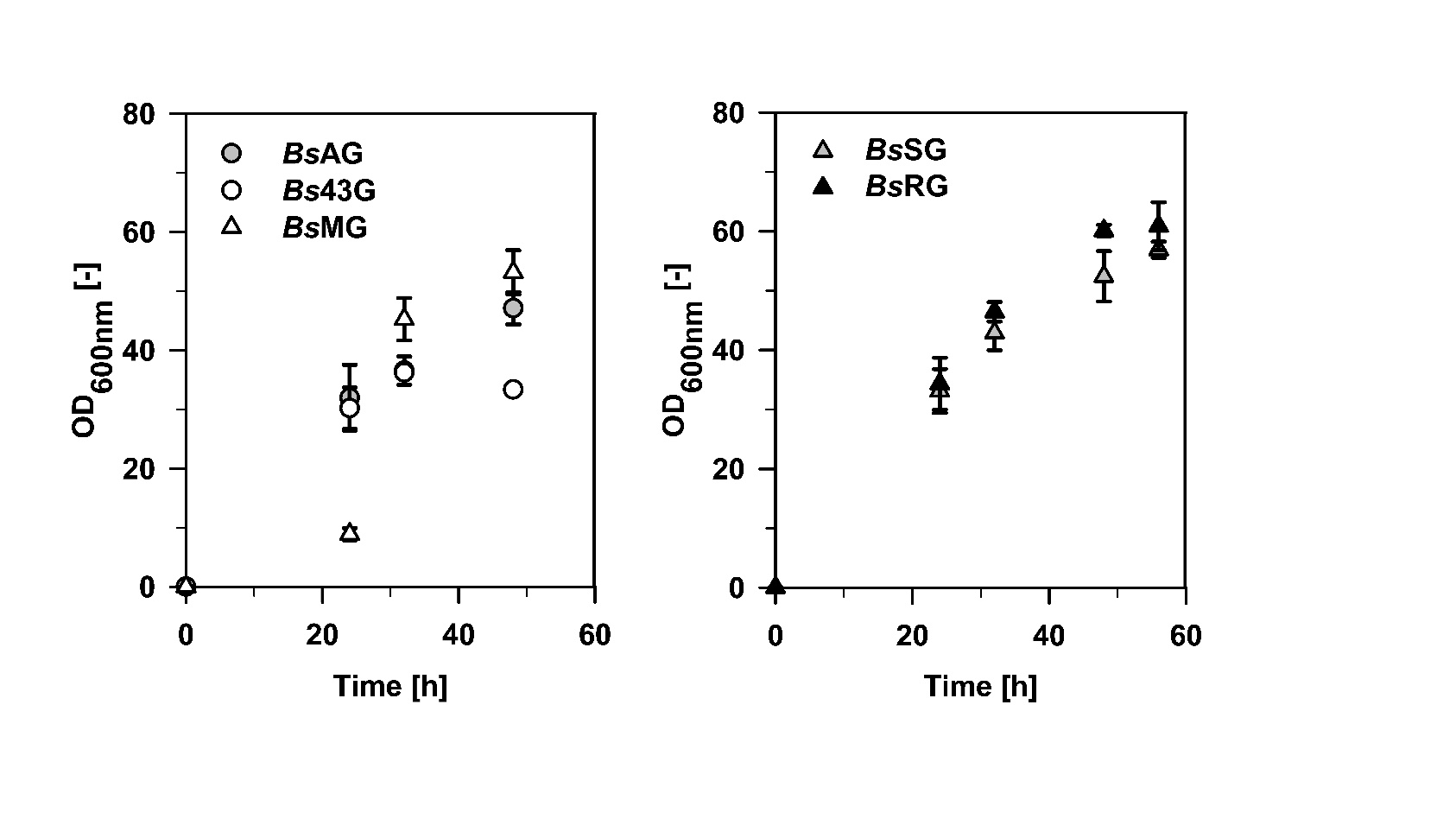


**Supplemental Figure 2: Growth curves of the recombinant *B. subtilis* strains for β-gal-Pw production.**


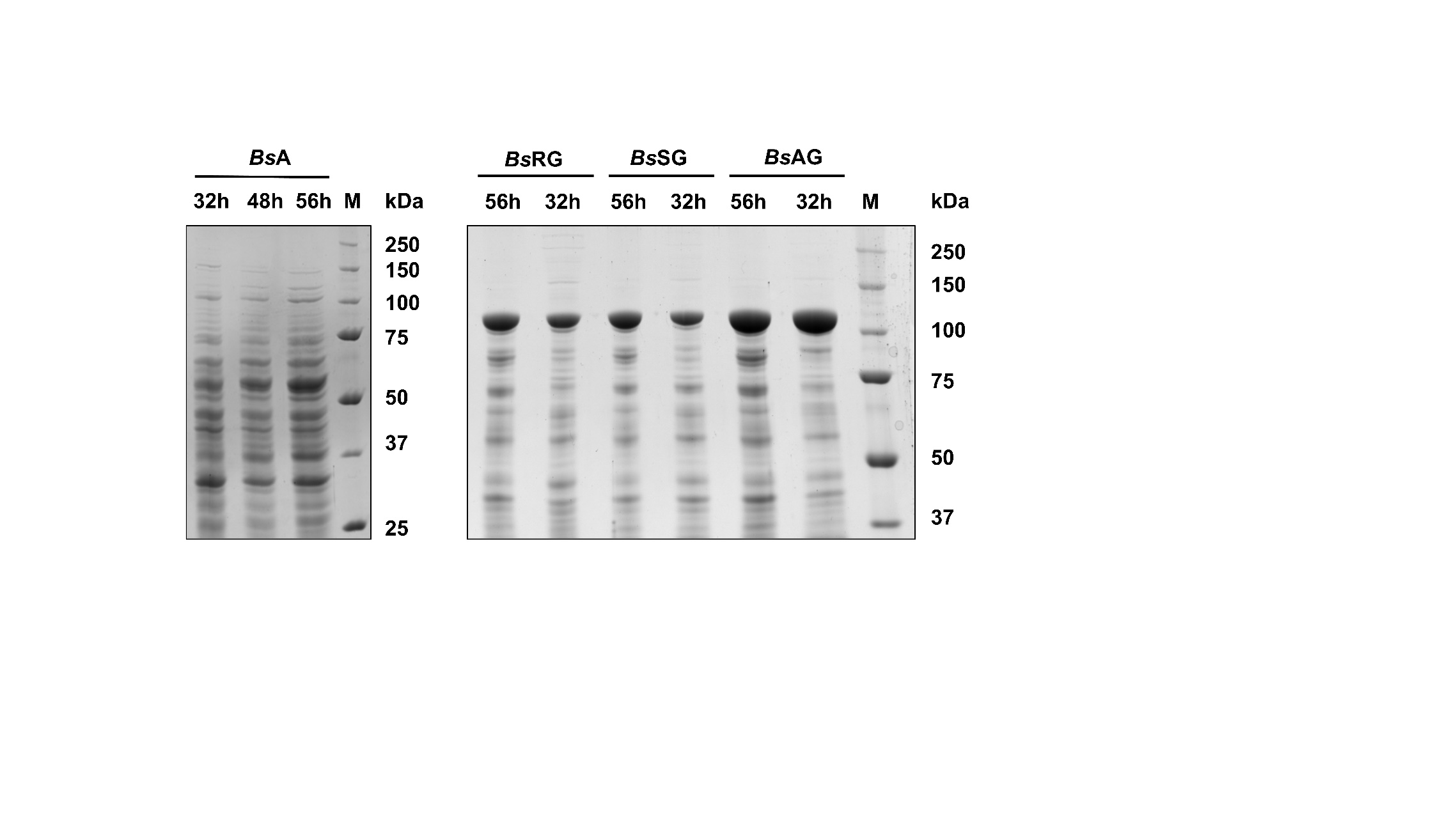


**Supplemental Figure 3: SDS PAGE of cell-free extract of recombinant *B. subtilis* strains.** Sampling was done after 32, 48 and 56 h of cultivation. An amount of 5 µg was loaded into each well. M = Marker


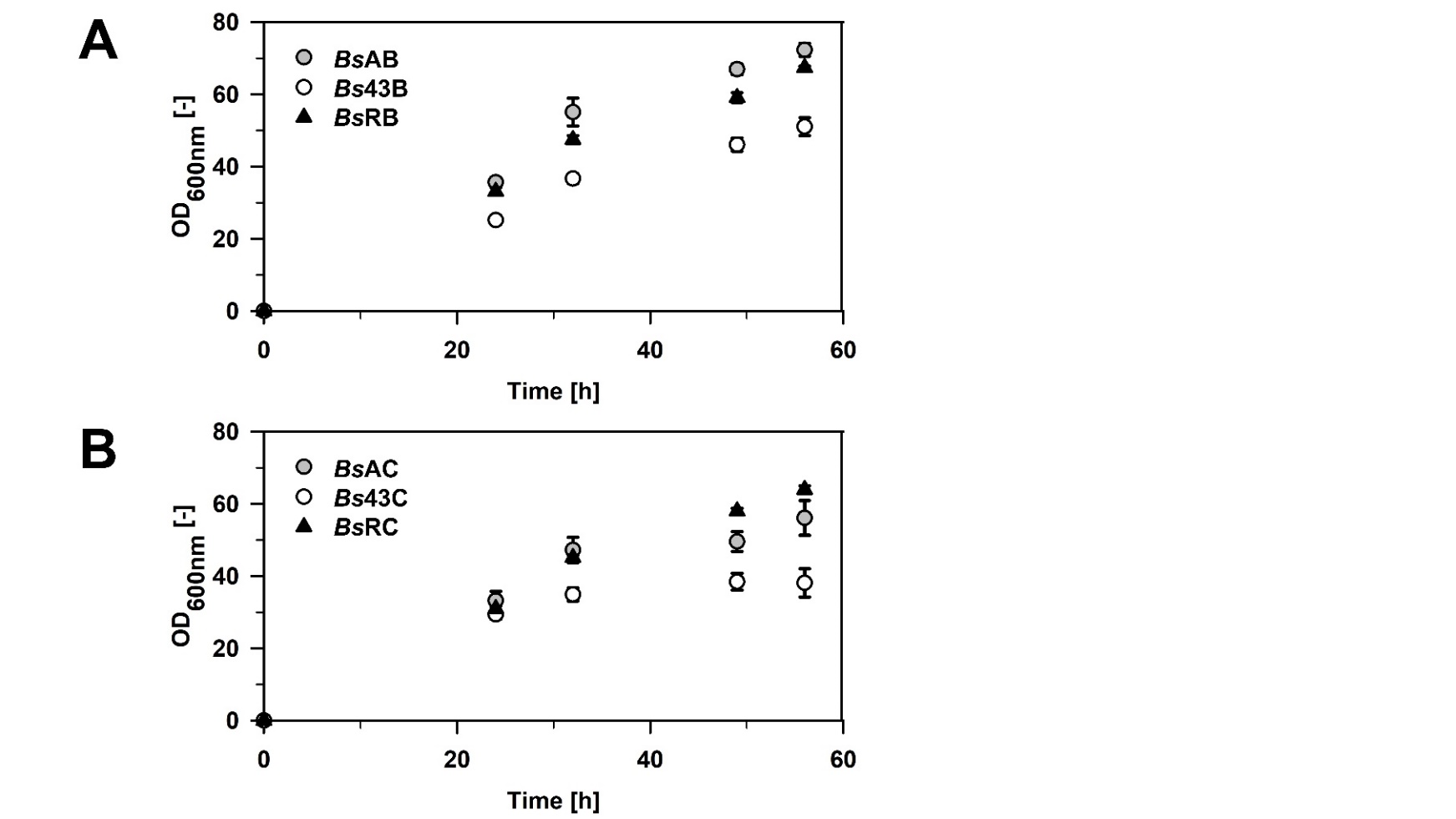


**Supplemental Figure 4: Growth of recombinant *B. subtilis* strains for the CelB (A) and CsCE (B) production.**


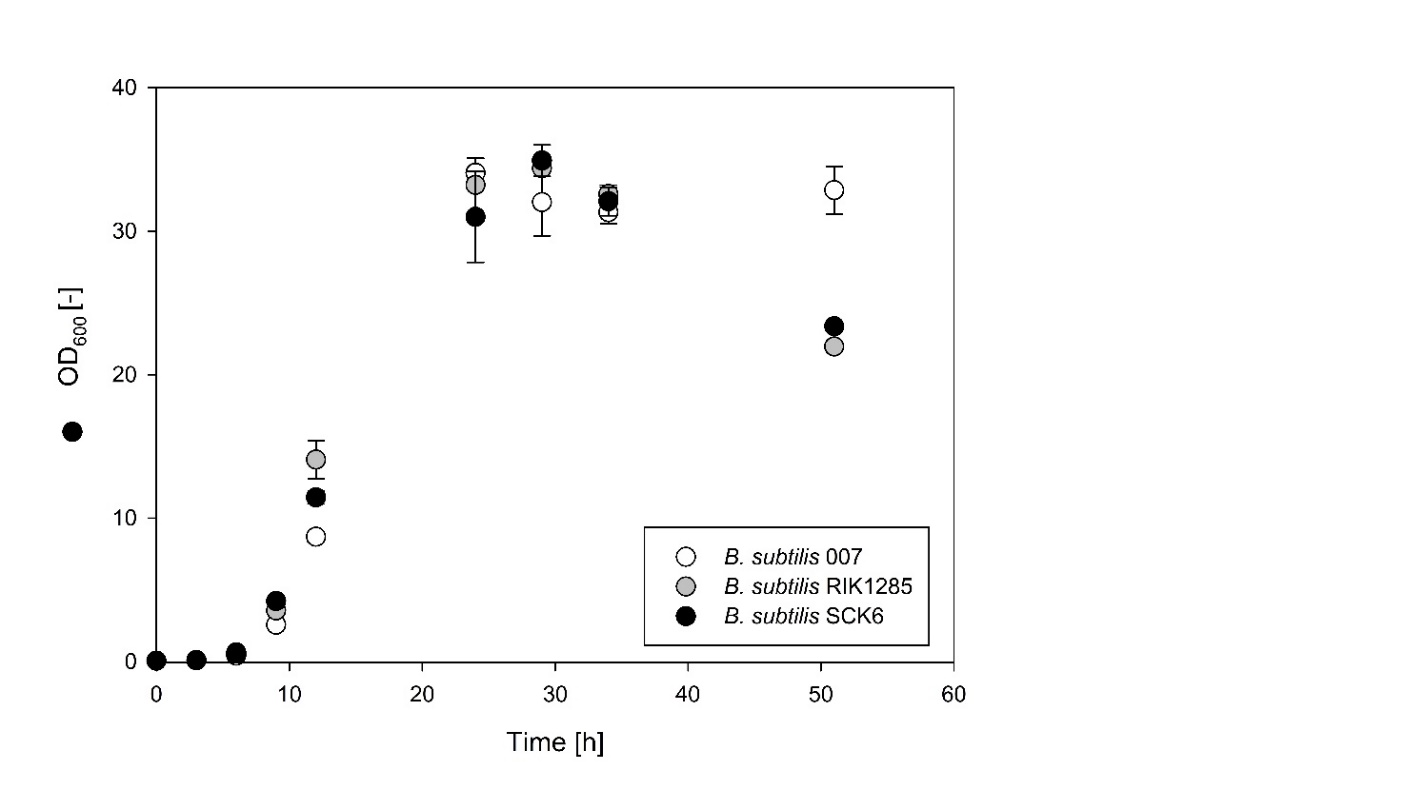


**Supplemental Figure 5: Growth of *B. subtilis* 007, RIK1285 and SCK6.** The shake flask cultivation was done in fermentation medium at 30 °C. *B. subtilis* RIK1285 (Takara Bio Inc.#3380) and SCK6 (Zhang et al. 2011) are domesticated *B. subtilis* 168 derivates.

**References**

Zhang XZ, Zhang YHP (2011). Simple, fast and high-efficiency transformation system for directed evolution of cellulase in *Bacillus subtilis*. Microbial Biotechnol 4:98–105. https://doi.org/10.1111/j.1751-7915.2010.00230.x
